# The Resistance of a North American Bat Species (*Eptesicus fuscus*) to White-Nose Syndrome (WNS)

**DOI:** 10.1371/journal.pone.0113958

**Published:** 2014-12-01

**Authors:** Craig L. Frank, Andrew Michalski, Anne A. McDonough, Marjon Rahimian, Robert J. Rudd, Carl Herzog

**Affiliations:** 1 Department of Biological Sciences, Fordham University, Louis Calder Center, P.O. Box 887, Armonk, NY 10504, United States of America; 2 Environmental Science Program, Fordham University, LH 400, Bronx, NY 10458, United States of America; 3 Wadsworth Center, New York State Department of Health, Albany, NY 12201, United States of America; 4 New York State Department of Environmental Conservation, 625 Broadway, Albany, NY 12233, United States of America; CSIRO, Australia

## Abstract

White-nose Syndrome (WNS) is the primary cause of over-winter mortality for little brown (*Myotis lucifugus*), northern (*Myotis septentrionalis*), and tricolored (*Perimyotis subflavus*) bats, and is due to cutaneous infection with the fungus *Pseudogymnoascus* (*Geomyces*) *destructans* (*Pd*). Cutaneous infection with *P. destructans* disrupts torpor patterns, which is thought to lead to a premature depletion of body fat reserve. Field studies were conducted at 3 WNS-affected hibernation sites to determine if big brown bats (*Eptesicus fuscus*) are resistant to *Pd*. Radio telemetry studies were conducted during 2 winters to determine the torpor patterns of 23 free-ranging *E. fuscus* hibernating at a site where *Pd* occurs. The body fat contents of free-ranging *E. fuscus* and *M. lucifugus* during hibernation at 2 different WNS-affected sites were also determined. The numbers of bats hibernating at the same site was determined during both: a) 4–7 years prior to the arrival of *Pd*, and, b) 2–3 years after it first appeared at this site. The torpor bouts of big brown bats hibernating at a WNS-affected site were not significantly different in length from those previously reported for this species. The mean body fat content of *E. fuscus* in February was nearly twice that of *M. lucifugus* hibernating at the same WNS-affected sites during this month. The number of *M. lucifugus* hibernating at one site decreased by 99.6% after *P. destructans* first appeared, whereas the number of *E. fuscus* hibernating there actually increased by 43% during the same period. None of the *E. fuscus* collected during this study had any visible fungal growth or lesions on their skin, whereas virtually all the *M. lucifugus* collected had visible fungal growth on their wings, muzzle, and ears. These findings indicate that big brown bats are resistant to WNS.

## Introduction

White-nose Syndrome (WNS) is an emergent disease that is estimated to have killed over 5,000,000 bats in the eastern USA and Canada. WNS was first observed at a single cave in New York State during the winter of 2006–2007, and has since spread to over 190 bat hibernation sites located in 25 U.S. states and 5 Canadian provinces. WNS causes large increases in over-winter mortality for at least 4 of the 6 bat species that over-winter in the northeast: little brown (*Myotis lucifugus*), northern (*Myotis septentrionalis*) Indiana (*Myotis sodalis*) and tricolored (*Perimyotis subflavus*) [1]. The white fungus associated with WNS has been identified as *Pseudogymnoascus* (formerly *Geomyces*) *destructans* (*Pd*), and it grows on the muzzle, wings, and ears of afflicted bats during hibernation [2],[3]. Histological analyses of affected *M. lucifugus, M. septentrionalis*, and, *P. subflavus* revealed that fungal hyphae penetrate both the epidermis and dermis, replacing hair follicles, sebaceous and sweat glands [2],[4]. Laboratory experiments have demonstrated that cutaneous infection with *P. destructans* is the cause of death in bats affected with WNS [5].

Hibernating bat species in NY congregate near caves/mines during mid-August, with the onset of hibernation by mid-October. Bats emerge from hibernation during April/May. During late summer/early fall, the body fat content of little brown bats (*M. lucifugus*) increases from 7 to 27% body mass [6],[7]. Fat is the primary energy source utilized during mammalian hibernation [8],[9]. Mammalian torpor is a state when body temperature (T_b_) and metabolic rate are both greatly reduced. It involves the regulation of T_b_ at a new and substantially lower level, with a new critical minimum T_b_ maintained [10]. Metabolic rates during torpor can be <5% of normothermic levels [8] with a minimum T_b_ as low as −2°C [11]. Hibernators do not remain torpid throughout the hibernation season; instead bouts of torpor last from days to weeks, interrupted by brief (<3 h for bats) periods of high metabolic rates and T_b_, known as arousal episodes [10]. Arousal episodes account for 80–90% of all energy (depot fat) utilized during hibernation, but their physiological function is unknown [8],[12]. Field studies indicate that cutaneous infection with *P. destructans* causes mortality through the disruption of normal torpor patterns during hibernation, causing more frequent arousal episodes, which is thought to lead to a premature depletion of body fat reserves prior to the availability of food, and subsequent death [13]. Laboratory hibernation experiments with *M. lucifugus* have confirmed that cutaneous infection with *P. destructans* causes the short torpor bouts characteristic of WNS [14].

A total of 47 bat species are found in North America, and more than half hibernate during winter [15]. It is unknown if all hibernating bats species found in North America are highly susceptible to cutaneous infection with *P. destructans* and subsequent WNS, however. There is considerable evidence that the big brown bat (*Eptesicus fuscus*) is highly resistant to cutaneous infection with *P. destructans*. Histological analyses of *E. fuscus* from WNS affected sites revealed no signs of fungal infection, whereas identical analyses performed on *M. lucifugus, M. septentrionalis*, and *P. subflavus* collected simultaneously from the same sites all showed extensive cutaneous infections with *Pd* [2]. The mean % body fat content of *E. fuscus* hibernating during February was nearly twice that *M. lucifugus* found in the same caves and mines and cutaneous infections with *P. destructans* were observed in *M. lucifugus*, but not *E. fuscus* [16]. Field studies conducted in New Hampshire during the active season (May – August) from 2005 through 2011 demonstrated 68–98% reductions in the capture rates by mist net for *M. lucifugus*, *M. septentrionalis*, and M. *leibii*, whereas the capture rate for *E. fuscus* did not significantly decrease during this period [17]. We thus propose that *E. fuscus* is highly resistant to WNS. Specifically, we predict that *E. fuscus* hibernating in areas where *P. destructans* occurs would: a) have torpor bouts of normal duration, b) emerge from hibernation with depot fat, and, c) show few signs of cutaneous infection with *P. destructans* during hibernation. These predictions were tested by examining the torpor patterns, body fat contents, wing skin, and numbers of *E. fuscus* hibernating at WNS affected sites in New York State. We also tested the hypothesis that the frequent arousal from torpor caused by cutaneous infections in WNS-affected bats leads to a premature depletion of depot fat reserves by simultaneously measuring the body fat contents and population sizes of *M. lucifugus* at our study sites.

## Materials and Methods

### Torpor Patterns of Hibernating Bats

This study was conducted in strict accordance with recommendations listed in the Guide for the Care and Use of Laboratory Animals of the National (US) Institutes of Health. The protocols were approved by the Fordham University Institutional Animal Care and Use Committee (protocol numbers CF11-03 and 12-01). Protocols were also approved by the New York State Department of Health Institutional Animal Care and Use Committee. The capture of live bats was also conducted under a Scientific License to Collect (#1373) issued by the New York State Department of Environmental Conservation.

This study was conducted at the Williams Lake Mine located in Ulster County, NY. *P. destructans* first appeared at this hibernation site during the winter of 2007–08. Sixteen adult *E. fuscus* (8 male, 8 female) were collected while torpid in this mine on 23 December 2011. Immediately upon collection, each bat was fitted with a temperature sensitive radio transmitter (Holohil model LB-2NT) and released back at the roost site within 30 min. Each transmitter was adhered to a shaved area on the back, between the shoulders (scapulas). Each transmitter weighed only 0.75 g, and had a battery of life ∼70 d. The skin temperature (T_skin_) of each bat was measured and recorded at 10–15 min. intervals throughout the study period by constantly monitoring radio signals using a computerized radio receiving system (ATS model 4500s) housed at this site. Skin temperature (T_skin_) is equivalent to body temperature (T_b_) in small bats [18]. Torpor was defined as when T_skin_ <23°C. Two antennas were placed inside each mine at areas where *E. fuscus* were roosting during hibernation. These antennas were connected to 50 Ω cables that ran to the mine entrance, and interfaced with the radio receiving system. This permitted weekly data downloads from the receiving system without re-entering the mine and disturbing hibernating bats. Ten more adult *E. fuscus* (all male) were collected while torpid at the Williams Lake Mine on 18 January 2013, and each was fitted with a temperature sensitive radio transmitter (Holohil model LB-2NT) as described previously. Radio signals were continuously monitored until all transmitter batteries were exhausted. The first (2012) group of bats was monitored until 26 March 2012, and the second group of bats was monitored until 29 March 2013.

Each of 26 *E. fuscus* captured was also screened for the wing lesions indicative of *Pd* infections and WNS using ultraviolet (UV) light prior to transmitter attachment following the techniques of Turner et al. [19]. All were kept in the mine, and both wings were extended, while being transilluminated by a portable long-wave (365–385 nm) UV light. This technique causes an orange-yellow fluorescence of the cupping erosions (lesions) due to *Pd* infection that is visible to the naked eye, and correctly indicates the presence of these lesions with an accuracy of 98% [19]. The presence or absence of an orange-yellow fluorescence was thus recorded for each wing. The ambient temperature (Ta) at which *E. fuscus* were hibernating during the first (2012) season was measured by placing an Onset model “Tidbit v2” temperature logger at a known *E. fuscus* roost site in this mine on 23 December 2011. It was programmed to record temperatures at 1 h intervals.

**Figure 1:**
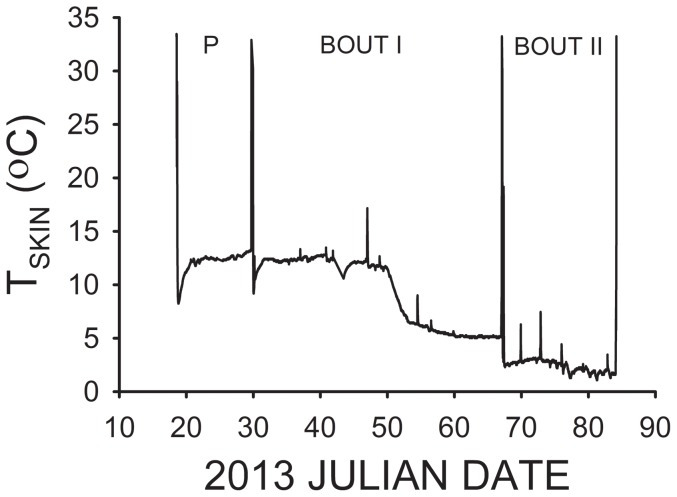
T_skin_ recordings of an individual male *E. fuscus* during hibernation. Torpor is when T_skin_ <23°C. The torpor bout labeled “P” is that which occurred after the arousal episode that was provoked by the capture and handling of this bat. Two subsequent natural torpor bouts occur after normal (spontaneous) arousal episodes. The bat arouses from torpor and leaves the mine at the end.

Previous studies on free-ranging *E. fuscus* revealed that while hibernating in caves, torpor bouts averaged 7–25 d in length [20]. The mean torpor lengths observed for each group of bats were thus statistically compared to 7 and 25 d to determine if they deviated from the normal torpor bout length previously reported for this species. Handling torpid bats for transmitter attachment caused them to quickly arouse, and they re-entered torpor within 30 min. upon their subsequent release (Figure 1). The first bout of torpor observed for each bat was therefore not included in the analyses of torpor patterns, in order to avoid any biases due to artifacts stemming from this preceding “provoked” arousal episode. Only T_skin_ data collected after the first spontaneous (natural) arousal episode recorded from each bat were included in the torpor pattern analyses. Mean T_skin_ and torpor bout lengths were compared among all groups for the first torpor bout observed using a one-way ANOVA (General Linear Models) procedure in conjunction with Tukey's Highly Significant Difference (HSD) Test. Mean T_skin_ and torpor bout lengths were compared between the 2 bat groups for the second torpor bout recorded using a Student's *t*-test. Comparisons of first and second recorded torpor bout parameters exhibited by the same group of bats were performed using a Paired *t*-test. All statistical methods were performed using SYSTAT version 12.0 software.

### Body Fat Levels of Free-Ranging Bats

Previous field studies on the torpor patterns of WNS-affected *M. lucifugus* revealed that cutaneous infections with *P. destructans* vary in degree of severity within the same hibernating population. Those *M. lucifugus* with the most severe *P. destructans* infections have torpor bouts that are 50–60% shorter in length than normal, and usually die well before the end of hibernation (late April in NY). The *M. lucifugus* with moderate *P. destructans* infections, however, usually have torpor bouts that are normal in length, and typically survive to the end of hibernation [13]. We thus collected bats at 2 different WNS-affected hibernation sites during 2 different periods: mid-hibernation (January-February) and natural emergence from hibernation (late April), in order to assess the body fat levels associated with these 2 degrees of cutaneous infection in *M. lucifugus*. Four adult *M. lucifugus* and 4 adult *E. fuscus* were collected from the Williams Preserve Mine located in Ulster County, NY, during mid-hibernation (January- February 2008), while torpid. All *M. lucifugus* were displaying signs of WNS with visible fungal hyphae on their ears, nose, and wings, whereas there we no visible signs of fungal infection on any of the *E. fuscus* collected. Eleven more adult *M. lucifugus* and 4 additional adult *E. fuscus* were collected from another mine located in Essex County NY on 24 April 2008, as they emerged from it at the end of hibernation. Most of these *M. lucifugus* had some fungal hyphae visible on their wings and muzzles, whereas none of *E. fuscus* collected had any visible signs of cutaneous infection. During each collection period, all bats were sacrificed immediately upon capture using a Isoflurane overdose, and their carcasses were frozen at −20°C until analysis. Adult *M. lucifugus* and *E. fuscus* are sometimes infected with rabies [21] which can also lead to rapid body fat loss during hibernation, thus all bat carcasses were first tested for rabies following the methods summarized in Blanton et al. [22]. None tested positive for rabies, thus all were included in the analyses.

Each carcass was first weighed to the nearest 0.0001 g, and dried at 60°C for 24 h. The dried carcass was then weighed again, homogenized, and dried again at 60°C for another 24 h. All lipids were subsequently extracted from the homogenized carcass in a Soxhlet apparatus for 12 h using petroleum ether following the techniques of Dobush et al. [23] to determine total lipid content. Chemical lipid extraction methods remove both depot and structural lipids, and do not distinguish between them. The structural lipids in mammalian tissues are phospholipids, cholesterol, ceramides, and sphingolipids, and none are either stored or mobilized as metabolic energy sources. Mammalian depot lipids, in contrast, are triacylglycerols which are stored in adipocytes, and they are both mobilized and catabolized during fasting [24]. Total body fat content (% live mass) thus represents the sum of both depot lipids, which can be mobilized to support metabolism during fasting, and structural lipids, which cannot. Mammals that have depleted all of their depot lipid reserves will consequently still have total body fat contents of 4–8% live mass [25], composed entirely of structural lipids. The mean total body fat contents obtained for both *M. lucifugus* and *E. fuscus* groups were thus compared to the minimum body fat contents reported for each of these species during normal summer lactation in order to estimate whether or not all depot lipids have been depleted. The field metabolic rate of bats is the greatest during lactation, and consequently depot fat levels are depleted. Virtually all body fats are structural lipids during this period. The minimum body fat content for *E. fuscus* previously reported was 5.5% body mass, whereas the lowest body fat content measured for *M. lucifugus* is 6.7% body mass [7],[26].

### Annual Bat Surveys

The total number of bats hibernating in the Williams Lake Mine during 5 different years was determined by visually counting all torpid bats roosting on the ceiling and walls of this mine during a single day-long survey each winter. Surveys were conducted by wildlife biologists from the NY State Department of Environmental Conservation (DEC) during the February-March periods of 2000, 2001, 2003, prior to the appearance of WNS, and during 2009 and 2010, after WNS arrived at this mine. The New York State Department of Health tests the carcasses of wild mammals submitted for rabies examination following exposure to either a person or domesticated animal. Almost all hibernating bat species submitted for testing are collected during their active (non-hibernation) seasons. The number of individual bats of a particular species submitted for testing during a given year should thus be positively correlated with the abundance of that species during the active season. The numbers of *M. lucifugus* and *E. fuscus* submitted to the Rabies Laboratory at the Wadsworth Center for testing from 2000 through 2012 were thus analyzed for any significant changes since the first appearance of WNS during the winter of 2007–2008, in order to provide insights into possible changes in the relative abundance of each bat species.

## Results

### Torpor Patterns of Hibernating Bats

**Figure 2:**
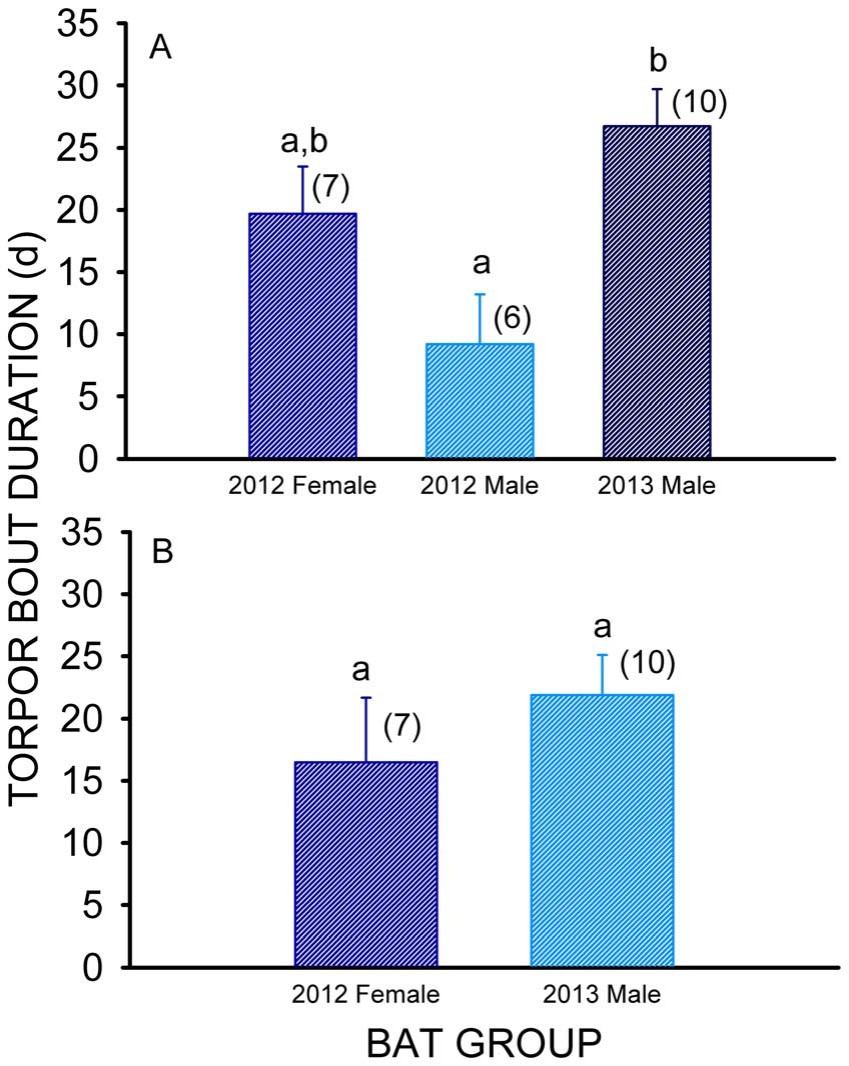
Mean (+ SE) durations of natural torpor bouts during hibernation by *E. fuscus*. First natural torpor bouts observed that occurred after normal arousal episodes during 2012 and 2013 (A) and second natural torpor bouts observed that occurred after normal arousal episodes during 2012 and 2013 (B). Means within the same torpor bout category sharing a common lower-case letter are not significantly different at the p<0.05 level, sample sizes (N) are given in parentheses.

The lengths of natural (not after a provoked arousal episode) torpor bouts observed for *E. fuscus* during 2012 ranged from 5.4 to 34.2 d, and varied from 10.2 to 41.9 d during 2013. Only 1–2 natural torpor bouts were thus measured for most bats prior to the depletion of the transmitter battery. A complete set of T_skin_ recordings for a single male *E. fuscus* during 2013 are summarized in Figure 1. Continuous T_skin_ recordings for 13 out of the 16 bats fitted with transmitters were obtained during 1 January-26 March 2012 (7 female, 6 male), and for all 10 male *E. fuscus* during 18 January-29 March 2013. The mean length of the first (January-February) natural torpor bout recorded for male *E. fuscus* during 2012 was less than half that observed for male *E. fuscus* during 2013 (F_2,18_ = 5.12, p = 0.017), but was not significantly different from the mean length of the first torpor bout observed for female *E. fuscus* during 2012 (Figure 2A). Likewise, the mean length of the first torpor bout observed for male *E. fuscus* during 2012 was not significantly different from the minimum of 7 d previously reported for this species (t = 0.55, df = 3, p = 0.62), but was significantly less that the maximum of 25 d previously reported for normal *E. fuscus* (t = −3.97, df = 3, p = 0.029). In contrast, the mean length of the first torpor bout measured for male *E. fuscus* during 2013 (Figure 2B) was significantly greater than 7 d (t = 6.74, df = 9, p<0.001), and statistically equivalent to 25 d (t = 0.59, df = 9, p = 0.57). The mean length of the first torpor bout observed for female *E. fuscus* (in 2012) was significantly greater than 7 d (t = 3.48, df = 6, p = 0.013, and not significantly different from 25 d (t = −1.56, df = 6, p = 0.17) as well.

Radio contact with most (4 out of 6) male *E. fuscus* was lost after the first torpor bout recorded in 2012, thus only the data on the second (February-March) recorded torpor bouts for 2012 females (N = 5) and 2013 males (N = 10) were analyzed (Figure 2B), and the mean lengths of the second torpor bout did not significantly differ between these two groups (t = −0.937, df = 13, p = 0.37). Furthermore, the mean length of the second torpor bout observed for female *E.fuscus* did not significantly differ from either 7 d (t = 1.84, df = 4, p = 0.14) or 25 d (t = −1.64, df = 4, p = 0.18). In contrast, the mean length of the second torpor bout observed for male *E. fuscus* in 2013 was significantly greater than 7 d (t = 4.70, df = 9, p = 0.001) but not significantly different from either 25 d (t = −0.97, df = 9, p = 0.36) or the mean length of the first 2013 torpor bout (t = 0.90, df = 9, p = 0.39).

Mean skin temperatures during the first torpor bouts recorded during 2012 and 2013 (Table 1) were not statistically different across all bat groups (F_2,18_ = 0.349, p = 0.71). The mean T_skin_ for female *E. fuscus* during the first and second torpor bouts recorded during 2012 (Table 1) did not significantly differ from each other (Paired t-test: t = 1.879, df = 3, p = 0.16). The mean T_skin_ for the first torpor bout of male *E. fuscus* recorded during 2013 (Table 1) was significantly greater than that of the second torpor bout recorded for this group (Paired t-test: t = 4.279, df = 9, p = 0.002). The first arousal episodes from torpor for male and female *E. fuscus* during 2012 (Table 1) did not significantly differ in mean duration (t = 0.249, df = 8, p = 0.81). The mean durations of the first and second arousal episodes observed during 2012 for female *E. fuscus* (Table 1) were also statistically equivalent (Paired t-test: t = 1.389, df = 3, p = 0.259). The mean durations of the first and second arousal episodes observed during 2013 for male *E. fuscus* were also statistically equivalent (Paired t-test: t = −0.961, df = 7, p = 0.368). No lesions were found on the wings of any of the 26 *E. fuscus* examined using long-wave UV light. The mean (± SE) ambient temperature (T_a_) at the roost site was 3.4±0.01°C during 2012, and ranged from 2.0 to 4.7°C during the study period.

**Table:** **Table 1.** Mean (± SE) skin temperatures during torpor and durations of arousal episodes for hibernating *E. fuscus*.

Year	Torpor Bout	Gender	N	T_skin_ (°C)	Arousal Episode (min)
2012	First Recorded	Female	7	13.3±1.1	116±54
		Male	6	13.2±1.4	102±19
	Second Recorded	Female	7	12.1±0.6	42±5
2013	First Recorded	Male	10	12.4±0.6	145±36
	Second Recorded	Male	19	7.5±1.4*	213±72

*Significantly different from the first torpor bout recorded for the same bat group at the p<0.05 level.

### Body Fat Levels of Free-Ranging Bats

The mean body fat content of *E. fuscus* collected during mid-hibernation was significantly greater than that of *M. lucifugus* collected during the same period (t = 3.985, df = 6, p = 0.007), by almost 2-fold (Table 2). The *E. fuscus* collected during emergence from hibernation had a mean body fat content that was significantly greater (Table 2) than that of the *M. lucifugus* collected at this site the same day (t = 1.656, df = 13, p = 0.032). The mean body fat content of *E. fuscus* collected during the mid-hibernation period was almost twice that of *E. fuscus* collected during emergence from hibernation (Table 2), but both groups had a mean body fat content that was significantly above the summer minimum of 5.5% body mass previously reported for this species (t = 5.821, df = 3, p = 0.005, and, t = 6.077, df = 3, p = 0.004, respectively) by Hood et al. [26]. The mean body fat content of *M. lucifugus* collected during mid-hibernation, as well as that of the *M. lucifugus* emerging from hibernation on 24 April 2008, were each statistically equivalent to the summer minimum body fat content of 6.7% (t = 0.74, df = 3, p = 0.51, and, t = −0.55, df = 10, p = 0.60, respectively) that was previously reported for this species by Reynolds & Kunz [7].

**Table:** **Table 2.** Mean (± SE) body fat contents of free-ranging *E. fuscus* and *M. lucifugus*.

Bat Species	Mid-Hibernation	Emergence
*E. fuscus*	15.8±1.8*	8.4±0.5*
*M. lucifugus*	7.5±1.1	6.3±0.7

Body fat contents are calculated as % live mass.

*Significantly greater than the corresponding *M. lucifugus* mean at the p<0.05 level. N = 4 for all means except for the emergence mean of *M. lucifugus*, where N = 11.

### Annual Bat Surveys

**Figure 3:**
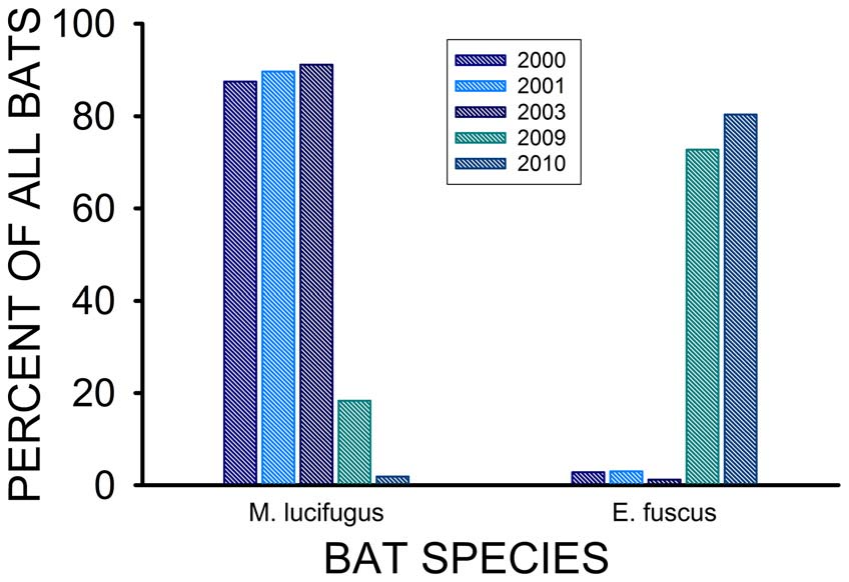
Relative proportions of all bats hibernating in the Williams Lake Mine that are *M. lucifugus* and *E. fuscus*. Values for the years of 2000, 2001, and 2003 represent conditions prior to the arrival of WNS in North America, whereas those for 2009 and 2010 represent those after the first appearance of WNS in this mine during the winter of 2007–08.

The total number of bats (all species combined) found hibernating in the Williams Lake Mine during February/March 2000, 2001, and 2003, was 5791, 6750, and 10,355, respectively. These hibernating communities were comprised of six different species: little brown (*Myotis lucifugus*), Indiana (*M. sodalis*), northern (*M. septentrionalis*), small footed (*M. leibii*), tricolored (*P. subflavus*), and big brown (*E. fuscus*) bats. During the 4–7 year period prior to the arrival of *P. destructans*, little brown bats (*M. lucifugus*) accounted for the majority of the bats hibernating in this mine (Figure 3), with 120–201 big brown bats constituting <3% of this group. Only 2–3 years after the arrival of *P. destructans* (March 2009 and 2010), the total number of bats hibernating in this mine decreased to 264 and 336, respectively, with *M. lucifugus* accounting for just 31 of the 264 bats found in 2009, and only 24 of the 336 bats counted during 2010. In contrast, 192 *E. fuscus* were found in this mine during the 2009 survey, and 270 were counted during 2010. Consequently, *E. fuscus* accounted for>70% of all hibernating bats after the appearance of *P. destructans* (Figure 3).

**Figure 4:**
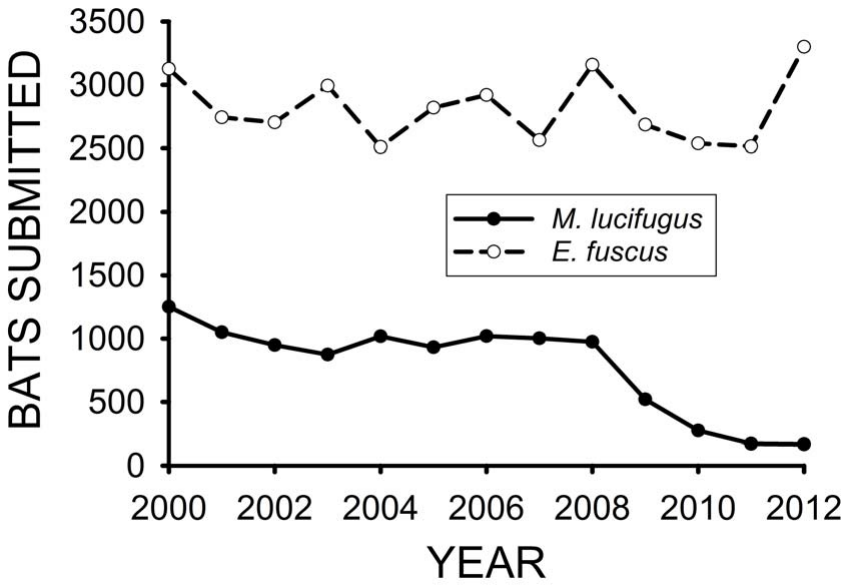
Numbers of *E. fuscus* and *M. lucifugus* submitted to the NYS Rabies Laboratory for testing from 2000 through 2012. WNS first became widespread in NY State during the winter of 2007–08.

The number of *E. fuscus* submitted to the NY State Rabies Laboratory per year was 2−10× the number of *M. lucifugus* received (Figure 4). The relative numbers of these species received, however, diverged to the greatest degree after the appearance of WNS. The mean (± SE) number of *M. lucifugus* submitted during 2000–2007 was 1102±40 bats/year (Figure 4), and it significantly decreased (t = −4.618, df = 11, p = 0.001) to 421±152 bats/year during 2008–2012. In contrast, the mean (± SE) number of *E. fuscus* submitted during 2000–2007 was 2797±75 bats/year, and did not significantly differ (t = 0.264, df = 11, p = 0.0796) from the mean of 2839±164 bats/year observed for the 2008–2012 period.

## Discussion

The results of our field studies support the hypothesis that big brown bats are highly resistant to WNS. The mean lengths of all torpors bouts observed for 23 *E. fuscus* hibernating at this WNS-affected site were all with the normal range of 7–25 d previously reported for this species by Brack & Twenty [20], and in most cases were not significantly different from 25 d. Big brown bats normally begin hibernation during November at latitudes similar to that of our study site [36]. Our measurements of T_skin_ did not begin until late December/early January, however, thus the lengths of the first and second torpor bouts observed in our study represent the typical torpor bout duration of *E. fuscus* during the middle of the hibernation season. The torpor bouts of *E. fuscus* hibernating at the Williams Lake Mine were normal in duration, as predicted. The mean body fat contents of *E. fuscus* collected from 2 other WNS-affected sites during both mid-hibernation, and at the end of hibernation, were significantly above the minimum (structural lipid) level of 5.5% body mass previously measured for this species by Hood et al. [26]. This indicates that the *E. fuscus* at these sites did not deplete all of their depot fat reserves prior to the end of hibernation, as predicted. The mean body fat levels of *M. lucifugus* collected from these same sites during these periods, however, were not significantly different from the minimum of 6.7% body mass recorded for this species by Reynolds & Kunz [7]. This indicates that *M. lucifugus* with either severe or moderate cutaneous infections with *P. destructans* completely deplete their depot fat energy stores prior to the end of hibernation. These findings support the hypothesis that frequent arousals from torpor caused by severe cutaneous infection with *P. destructans* produces a premature depletion of depot fat reserves since such infections have already been demonstrated to reduce the torpor bout lengths of *M. lucifugus* by 50–60% [13].

The transillumination of wings with long-wave UV light did not reveal any signs of the lesions indicative of WNS in the 26 hibernating *E. fuscus* examined at the Williams Lake Mine. The total number of big brown bats hibernating at this site not only remained relatively constant after *P. destructans* first appeared, but actually increased. In contrast, the number of little brown bats hibernating in this mine actually decreased by 99.6% during the same period (Figure 3).

It has been frequently assumed that *E. fuscus* in the Northeast is severely affected by WNS [27]. This assumption is apparently based on the findings of Turner et al. [1] where the analysis of visual counts for all bats hibernating at 42 sites where WNS now occurs revealed a 91–98% decrease in the number of *Myotis spp.*, and a 41% decrease in the number of *E. fuscus*, since the first appearance of *P. destructans*. This study did not determine the causes of the decreases observed, however. It thus appears that the decline reported for hibernating *E. fuscus* observed by Turner et al. [1] was due to ecological factors other than WNS.

Some other bat species have also been shown to be resistant to WNS as well. A survey of 366 bat hibernation sites in Europe revealed *P. destructans* growing on the muzzles of 21 individuals of 5 different European bats (*Myotis dasycneme*, *M. myotis*, *M. duabentonii*, *M. brandtii* and *M. oxygnathus*) during torpor. Mass deaths were not observed at these hibernation sites, however. These cutaneous infections are typically restricted to the outer epidermis (stratum corneum), and do not extend into the dermis as in North American *Myotis spp*. [28]. Furthermore, *M. myotis* with cutaneous *P. destructans* infections normally survive the hibernation period and persist into the next year [28],[29]. There is also some evidence that *M. myotis* with *P. destructans* growing on their muzzles during torpor do not prematurely deplete their body fat reserves [30]. It thus appears that some bat species have the ability to limit the degree of cutaneous infection with *P. destructans* during torpor to just the outer epidermis, thereby avoiding the lesions and subsequent short torpor bouts that lead to the premature depot fat depletion associated with WNS.

It is not fully understood how cutaneous infection with *P. destructans* causes a reduction in torpor bout length. The evaporative water loss (EWL) of bats is considerable during torpor, and it is thought that they periodically arouse to drink [31]. Hibernating bats have been recorded drinking during arousal episodes [32]. The skin lesions caused by *Pd* infections may increase the EWL of affected bats, which in turn would cause them to arouse from torpor more frequently to drink [33]. Analyses of blood samples collected from both *Pd* infected and uninfected *M. lucifugus* during hibernation support the hypothesis that *Pd* infections increase EWL [34],[35]. Further investigation of the resistance of big brown bats to *P. destructans* will provide new insights into how WNS kills hibernating bats, and will help to predict which bat populations/species are vulnerable to this devastating disease.
